# Supraglottic squamous cell carcinomas have distinctive clinical features and prognosis based on subregion

**DOI:** 10.1371/journal.pone.0188322

**Published:** 2017-11-20

**Authors:** Weiquan Ding, Tianrun Liu, Jiangang Liang, Tingbao Hu, Shaoyun Cui, Guorong Zou, Weiwei Cai, Ankui Yang

**Affiliations:** 1 Department Otorhinolaryngology, Panyu Central Hospital, Guangzhou, China; 2 Cancer Institute of Panyu, Guangzhou, China; 3 Department of Otorhinolaryngology Head and Neck Surgery, The Sixth Affiliated Hospital of Sun Yat-sen University, Guangzhou, China; 4 Department of Head and Neck Surgery, Sun Yat-sen University Cancer Center, Guangzhou, China; 5 State Key Laboratory of Southern China, Guangzhou, China; Technische Universitat Munchen, GERMANY

## Abstract

**Objective:**

To analyze the clinicopathologic characteristics and prognosis of patients with squamous cell carcinoma localized to different supraglottic subregions.

**Methods:**

Clinicopathologic data were reviewed retrospectively for 111 patients with supraglottic squamous cell carcinoma who were diagnosed between January 1, 1995 and December 31, 2005 and were initially treated with surgery. DNA from human papillomavirus (HPV) 16 and (or /and) HPV 18 were detected in all the 111 supraglottic carcinoma specimens using in situ hybridization. Survival analysis was performed by Kaplan-Meier analysis, factors were compared using log-rank test, and prognostic factors were determined using Cox proportional hazards model. The relationship between subregions and clinicopathologic factors was analyzed using Chi-square tests.

**Results:**

HPV prevalence differed between patients with aryepiglottic fold carcinoma and ventricle carcinoma (*P* < .05). The local-regional control rates, overall survival rates or cancer specific survival rates were significantly different between different subregions. Univariate analysis indicated that pTNM classification, pN spread, and subregion were associated with prognosis (*P* < .05). Multivariate analysis indicated that pTNM classification and subregion were associated with supraglottic carcinoma prognosis. The survival rate was better for patients with carcinoma of the epiglottis or ventricular bands compared to those with carcinoma in the aryepiglottic fold or ventricle (*P* = .012).

**Conclusions:**

Subregion may be a new prognostic factor for supraglottic squamous cell carcinoma. Different supraglottic carcinoma subregions have distinct clinical features such as HPV expression, lymph node metastasis rate, local-regional control and prognosis. Therefore, it is necessary to subdivide supraglottic squamous cell carcinomas into several subregion groups to individualize therapy.

## Introduction

Laryngeal carcinoma is one of the most common malignancies of the head and neck [1, 2]. Supraglottic carcinoma is the dominant laryngeal tumor, especially in some developing countries where alcohol and smoking are the most common risk factors [2, 3]. Patients with supraglottic carcinoma often present with non-specific throat pain, dysphagia, and neck lymphadenopathy as the primary disease features. These patients tend to present for care at an advanced stage and have poor overall survival [4, 5]. The supraglottic laryngeal region includes the epiglottis, ventricular bands, aryepiglottic fold (laryngeal aspect), and laryngeal ventricle, each of which serves a distinct anatomic purpose [6]. Until now, it has been unclear whether clinical features and prognosis differ based on carcinoma subregion.

In this study, supraglottic carcinoma was divided into four sub-types based on subregion: epiglottis, ventricular bands, aryepiglottic fold (laryngeal aspect), and laryngeal ventricle. Differences in clinicopathologic characteristics, HPV presence, and survival were then compared by subregion.

## Materials and methods

### Clinical data

The clinicopathologic data were analyzed retrospectively for 111 patients with supraglottic squamous cell carcinoma who were diagnosed between January 1, 1995 and December 31, 2005 and were initially treated with surgery at Sun Yat-sen University Cancer Center / Panyu Central Hospital (Table 1). We divided supraglottic squamous cell carcinomas into four types based on subregion: epiglottis, ventricular bands, aryepiglottic fold, and ventricle. The tumor location and diagnosis were established using clinical examination, fibro-laryngoscope, computed tomography, magnetic resonance imaging, intraoperative exploration, and postoperative histopathologic report. All patients underwent surgery for the primary lesion and selective or therapeutic neck dissection. Postoperative radiotherapy is advised in case of cartilage invasion, positive surgical margins, T3 or T4 tumors, advanced nodal disease (e.g. N2c/N3) with extracapsular invasion. Patients at high-risk for recurrence received post-operative radiotherapy based on NCCN (National Comprehensive Cancer Network) guidelines at a mean dose of 60 Gy (range, 40 Gy to 70 Gy). Three patients who developed distant metastases after surgery received chemotherapy with docetaxel, cisplatin, and fluorouracil. Clinicopathologic features are shown in Table 1. Pathologic diagnosis of squamous cell carcinoma was confirmed by two experienced pathologists.

**Table 1:** Univariate factors influencing prognosis of supraglottic squamous cell carcinoma.

Variable	Epiglottis	Ventricular bands	Aryepiglottic fold	Ventricle of larynx	*P*
**Sex**					
Men	25	22	26	33	0.858
Women	2	1	1	1	
**Age**					
<60 years	10	14	14	12	0.184
≥60 years	17	9	13	22	
**pT stage**					
T1	2	0	1	1	0.431
T2	9	11	6	11	
T3	13	9	11	13	
T4	3	3	9	9	
**pN stage**					
N0	13	16	17	28	0.119
N1	6	2	6	2	
N2	8	5	4	4	
N3	0	0	0	0	
**pTNM stage**					
Stage I	2	0	1	1	0.588
Stage II	5	8	3	11	
Stage III	11	8	13	11	
Stage IV	9	7	10	11	
**Histology grade**					
G1	10	9	6	12	0.260
G2	9	11	14	19	
G3	8	3	7	3	
**Surgery type**					
Supraglottic laryngectomy	1	3	1	1	0.000
Horizontal hemilaryngectomy	12	1	5	0	
Vertical hemilaryngectomy	0	7	0	11	
Frontolateral laryngectomy	2	0	0	0	
Total laryngectomy	12	12	21	22	
**R1 resection**					
Yes	5	5	6	6	0.964
No	22	18	21	28	
**Treatment modality**					
S	18	11	17	19	0.532
S+R	9	12	9	13	
S+C	0	0	1	2	
**Neck metastasis**					
Yes	13	16	17	28	0.042
No	14	7	10	6	

S, Surgery; R, Post-operative radiation therapy; C, Post-operative chemotherapy; R1 resection was defined when the distance of the tumour from the resection margin was less or equal 1 mm.

All patients were provided informed consent at the time of diagnosis between January 1, 1995 and December 31, 2005 for study participation using medical records and clinicopathologic data. Written ethics approval was obtained from the Hospital Research Ethics Committee of Panyu Central Hospital and Sun Yat-sen University Cancer Center.

### Detection of human papillomavirus using in situ hybridization

Formalin-fixed, paraffin-embedded tissue samples from 111 patients were sectioned serially at 5 mm thickness. After de-paraffinization, in situ hybridization (ISH) was used to detect HPV. Samples were hybridized with a biotinylated whole-genomic DNA probe mixture of HPV 16 and 18 (ISH-5121, Beijing Zhongshan Jingqiao Company, China). Cervical carcinoma was used as an ISH positive control and a reverse HPV probe in the hybridization mix was used as an ISH negative control. The experiments were performed from August 1, 2011 to December 31, 2011.

### Follow-up

Follow-up data were obtained by outpatient chart review and telephone interview during the period from the date of first treatment to December 31, 2013. The tumor-based end points included local-regional control, cancer-specific survival and overall survival.

### Statistical analysis

The data was analyzed using SPSS 16.0 software. Survival analysis was undertaken by Kaplan-Meier analysis; factors were compared using log-rank test, and multivariate analysis was carried out using Cox proportional hazards model (backwards-selection method). The relationship between different factors was analyzed using chi-square tests. *P* value less than .05 was considered statistically significant.

## Results

### Clinicopathologic characteristics of supraglottic carcinoma subregions

Lymph node metastasis rates were 48.1%, 69.6%, 63.0%, and 82.4%, respectively, for supraglottic squamous cell carcinomas of the epiglottis, ventricular bands, aryepiglottic fold, and ventricle. Although lymph node metastasis rates differed between subregions (*P* = .042), there were no correlations between subregion and age, sex, or histologic grade (Table 2). The incidence of R1-resection was 14.4% (16/111).

**Table 2:** Correlation between clinical factors and supraglottic squamous cell carcinoma subregions.

Variable	χ^2^	*P*
**Sex**	0.764	0.858
**Age**	4.837	0.184
**Histologic grade**	7.713	0.260
**Surgery type**	50.178	0.000
**Treatment modality**	5.091	0.532
**Neck lymph nodes metastasis**	8.815	0.042

### HPV presence by supraglottic carcinoma subregion

HPV was detected in 16 of 111 patients (14.4%) by ISH, but there was no correlation between HPV status and subregion (Table 3). When HPV status was compared for each pair of subregions, only the aryepiglottic fold and ventricle differed (χ2 = 50.565, *P* < .05).

**Table 3:** Clinicopathologic features for 111 patients with supraglottic squamous cell carcinoma and human papillomavirus.

Variable	Cases	HPV (-) N = 95 (85.6%)		HPV (+) N = 16 (14.4%)		*P*
		N1	%	N2	%	
Subregions						0.082
Epiglottis	27	23	85.2	4	14.8	
Ventricular bands	23	19	82.6	4	17.4	
Aryepiglottic fold	27	20	74.1	7	25.9	
Ventricle	34	33	97.1	1	2.9	

### Local-regional control rate

**Fig 1:**
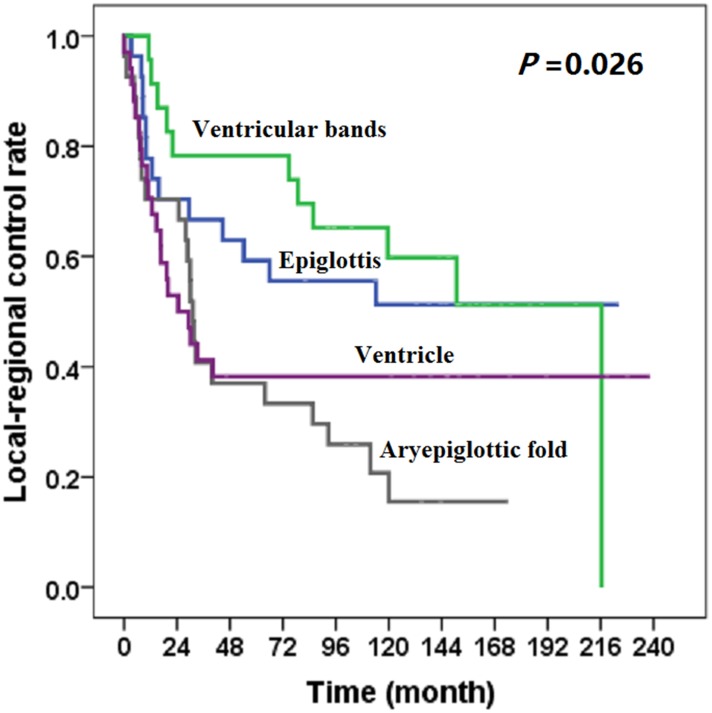
Local-regional control rate for patients with supraglottic squamous cell carcinoma, by subregion.

At the last follow-up date of December 31, 2013, 67 patients had local-regional recurrence. Subgroups differed with respect to local-regional control rate (χ2 = 9.235, *P* = .026) (Fig 1). The local-regional control rates of carcinoma from epiglottis or ventricular bands was better than that of carcinoma from aryepiglottic fold or ventricle (Fig 1). Carcinoma subregion was correlated with local-regional recurrence (Table 4).

**Table 4:** Local-regional control rate of 111 patients with supraglottic carcinoma.

Subregions	Cases	2-year (%)	5-year (%)	*P*
Epiglottis	27	70.4	59.3	0.026
Ventricular bands	23	78.3	78.3	
Aryepiglottic fold	27	70.4	37.0	
Ventricle	34	52.9	38.2	

### Survival

**Fig 2:**
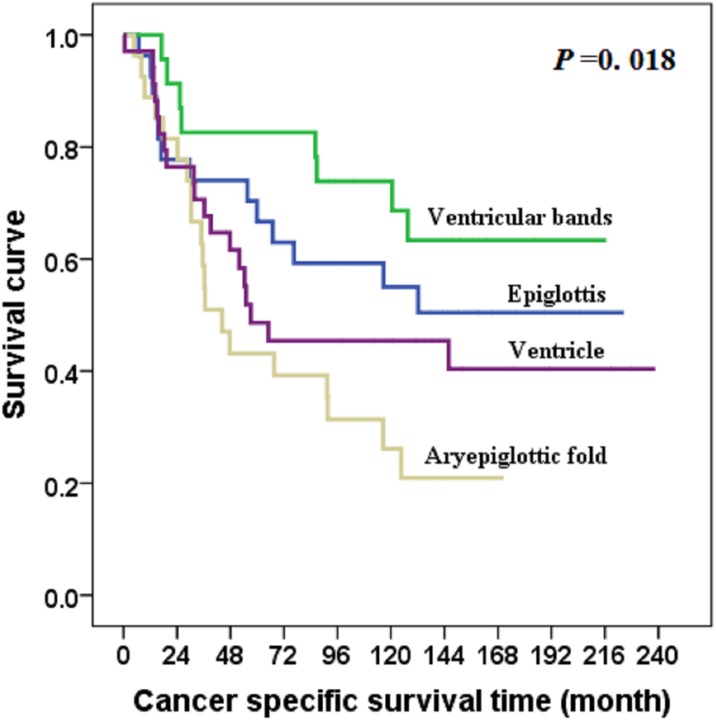
Cancer-specific survival for patients with supraglottic squamous cell carcinoma, by subregion.

**Fig 3:**
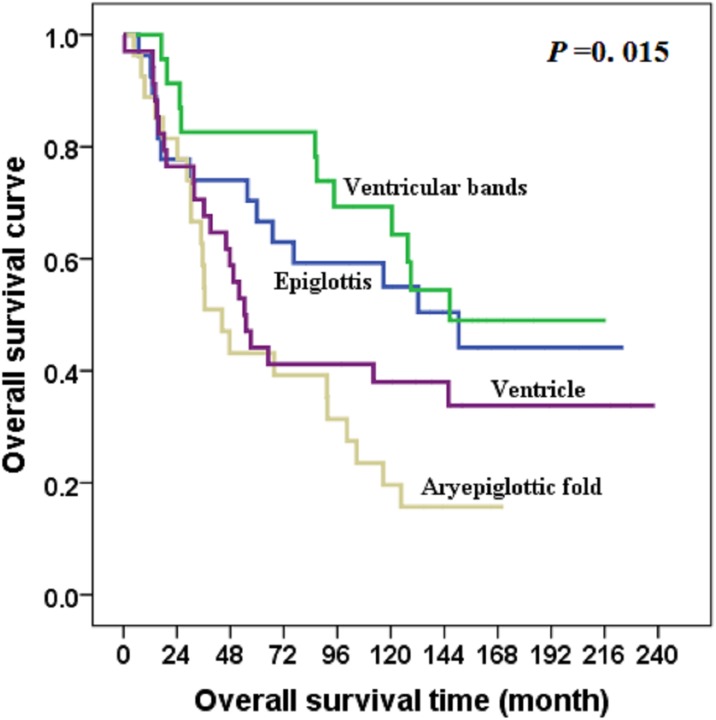
Overall survival for patients with supraglottic squamous cell carcinoma, by subregion.

At the last follow-up date of December 31, 2013, seven patients were lost to follow-up, and all patients had been followed for at least 2 years (median, 64 months). The overall survival rate was 42.3%, and 43 patients experienced tumor relapse (10 in primary site, 26 in cervical lymph nodes, two in primary site plus cervical lymph nodes, and three in distant site). Subregions were correlated with overall survival (Table 1). Subgroups differed with respect to cancer-specific survival (χ2 = 10.042, *P* = .018) and overall survival (χ2 = 10.526, *P* = .015) (Figs 2 and 3).

### Univariate analysis and multivariate analysis of prognostic factors

To determine which factors affect survival of patients with supraglottic carcinoma, possible prognostic factors of sex, age, subregion, stage, histologic grade, lymph node metastasis, surgery type, and treatment modality were analyzed by univariate analysis and multivariate regression analysis. Results from the univariate analysis showed that subregion, pN spread, and TNM classification were significantly correlated with overall survival (Table 5). Multivariate analysis results indicated that subregion and pTNM classification were independent prognostic factors affecting survival (Table 6).

**Table 5:** Factors influencing prognosis of patients with supraglottic squamous cell carcinoma.

Variables	Cases	5-year overall Survival rates	χ^2^	*P*
**Sex**			0.498	0.48
Men	106	41.5		
Women	5	60.0		
**Age**			0.049	0.824
<60 years	50	44.0		
≥60 years	61	41.0		
**Subregion**			11.049.	0.012
Epiglottis	27	51.9		
Ventricular bands	23	60.9		
Aryepiglottic fold	27	25.9		
Ventricle of larynx	34	35.3		
**pTstage**			7.325	0.062
T1	4	75.0		
T2	37	48.6		
T3	46	41.3		
T4	24	29.2		
**pN stage**			8.614	0.013
N0	74	45.9		
N1	16	50.0		
N2	21	23.8		
N3	0	0		
**pTNM stage**			10.880	0.012
Stage I	4	75.0		
Stage II	27	51.9		
Stage III	43	46.5		
Stage IV	37	27.0		
**Histology grade**			0.557	0.757
G1	37	43.2		
G2	53	39.6		
G3	21	47.5		
**Surgery type**			7.386	0.117
Partial supraglottic laryngectomy	6	16.7		
Horizontal hemilaryngectomy	18	50.0		
Vertical hemilaryngectomy	18	61.1		
Frontolateral laryngectomy	2	50.0		
Total laryngectomy	67	37.3		
**Treatment modality**			0.127	0.939
Surgery	65	43.1		
Post-op RT treatment	43	41.9		
Post-op chemotherapy	3	33.3		
**Neck lymph nodes metastasis**			2.961	0.085
Yes	37	35.1		
No	74	45.9		

**Table 6:** Factors influencing prognosis of supraglottic squamous cell carcinoma in multivariate analysis.

Factors	B	SE	Wald	*P*	Exp (B)	95.0% CI for Exp (B)
**pTNM**	0.455	0.199	5.216	0.022	1.576	(1.067–2.329)
**Subregion**	0.265	0.131	4.075	0.044	1.304	(1.008–1.687)

## Discussion

This study showed no differences with respect to patient age or sex, tumor histologic grade, or treatment modality and supraglottic carcinoma subregion. However, prognosis was better for patients with carcinoma of the epiglottis and ventricular bands compared to the aryepiglottic fold or ventricle, with survival rates of 51.9%, 60.9%, 25.9%, and 35.3%, respectively (*P* = .012). Because epiglottal and ventricular band carcinoma tend to grow anteriorly or downward and do not invade deep tissues until late in the disease, patients with these tumors may have better survival.

The supraglottic laryngeal region includes the epiglottis, ventricular bands, aryepiglottic fold (laryngeal aspect), and laryngeal ventricle, each of which serves a distinct anatomic purpose. For the purpose of tumor resection and function preservation, the surgical methods for carcinoma in each subregion is different, therefore, there appeared differences in the baseline distribution of surgical method between the different subregions, however, that might not lead to statistical bias. Patients with carcinoma of the epiglottis or aryepiglottic fold commonly undergo resection by horizontal hemi-laryngectomy or total laryngectomy, whereas those with carcinoma of the ventricular band or ventricle carcinoma usually undergo vertical hemi-laryngectomy or total laryngectomy [7–9]. There is a relatively wider space for epiglottal tumor growth before invasion to other subregions; thus, successful resection may be more feasible. Because the ventricular band and ventricle subregions are more limited in size, carcinomas in these two subregions may extend earlier in the longitudinal direction. Most patients with ventricular band or ventricle carcinoma had a poor prognosis, with stage III (43 of 111) or stage IV (37 of 111) disease at diagnosis. In this study, subgroups differed with respect to local-regional control rate (*P* < .05). The local-regional control rates of carcinoma in epiglottis or ventricular bands was better than that in aryepiglottic fold or ventricle, therefore, carcinoma in different subregions might present different local-regional rate after treatment.

There were also differences in neck lymph node metastasis rates between supraglottic carcinoma subregions (*P* = .045). Carcinomas of the aryepiglottic fold (37.0%) and epiglottis (51.9%) more often metastasized to neck lymph nodes than did carcinomas of the ventricular bands (30.4%) or ventricles (17.6%); this spread is due to the higher density of micro-lymphatic nets in aryepiglottic fold and epiglottis than that in the ventricular bands or ventricles.

As reported previously, HPV detection rates range from 26% to 44% in laryngeal cancers [10]. HPV 16 and 18 are detected at high levels in head and neck squamous cell carcinomas. HPV 16 accounts for 86.7% of the HPV-positive oropharyngeal squamous cell carcinomas, whereas HPV 18 accounts for only 2.9%. For HPV-positive oral squamous cell carcinomas, HPV 16 is found in 68.7% and HPV 18 is found in 34.5%. In all the HPV-positive laryngeal cancer cases, HPV 16 is detected in 69.2% of the cases and HPV 18 is detected in 17.2% of the cases. HPV-positivity rates are lower for squamous cell carcinomas of other areas of the head and neck [11].

HPV expression levels also differ by laryngeal subregion. Almadori et al found the HPV-positivity rate to be much higher in supraglottic carcinoma compared to other laryngeal areas [12]. For HPV-positive laryngeal squamous cell carcinoma, positivity rates are 60.6%, 36.4%, and 3.0% for the supraglottic, glottic, and subglottic regions, respectively [13]. HPV infection may be significantly correlated with laryngeal carcinoma subregion but not with clinical stage, lymph node metastasis, or tumor grade [13]. In our research, HPV expression was 14.7%; however, HPV expression was higher in ventricular band (25.9%), epiglottis (14.8%), and aryepiglottic fold (17.4%) carcinoma compared to ventricle carcinoma (2.9%). HPV expression differed between aryepiglottic fold and ventricle carcinoma (*P* = .008). Compared to ventricle carcinoma, other areas had higher HPV detection rates; those three subregions are nearer to the oropharynx, where carcinomas tend to have higher HPV expression rates [14]. Because supraglottic carcinomas require different surgical approaches based on subregion, HPV positivity, and prognosis, these tumors should be categorized by subregion to individualize therapy.

Demographic and clinical characteristics have been studied extensively with respect to prognosis of laryngeal carcinoma. As with previous studies, [4, 15, 16] demographic data such as age and sex were not associated with prognosis in our study. Raitiola and colleagues showed that T stage and cervical lymph node metastases are independent prognostic factors for laryngeal carcinoma [17]. Likewise, Santos et al found that T stage, N stage, clinical stage, and extracapsular lymph node spread were related to prognosis, with lymph node metastases independently affecting prognosis [18]. Similarly, Vlachtsis et al showed that T stage, N spread, TNM classification, and tumor site affect prognosis, with TNM classification independently predicting prognosis [19]. However, no studies to date have evaluated the significance of laryngeal carcinoma subregions in prognosis. In our study, carcinoma subregion, pN spread, and pTNM classification had prognostic significance in univariate analysis, with subregion and pTNM classification as independent predictors of prognosis.

Our study showed that different subregions of supraglottic carcinoma were associated with distinct clinical features such as HPV expression, lymph node metastasis rate, local-regional control and prognosis. Therefore, supraglottic squamous cell carcinoma should be considered as distinct subtypes corresponding to their subregions of origin so that patients can receive individualized therapy. However, some risk factors like alcohol and smoking are not included in the analysis due to the incomplete data, which may affect the results, and the number of cases in this study is relatively small, further studies would be needed to confirm that different subregions of supraglottic carcinoma show distinct clinical features.

## Supporting information

S1 Dataset Data subtype brief for PLOS 2011. (XLSX)
